# *phenix.model_vs_data*: a high-level tool for the calculation of crystallographic model and data statistics

**DOI:** 10.1107/S0021889810015608

**Published:** 2010-05-22

**Authors:** Pavel V. Afonine, Ralf W. Grosse-Kunstleve, Vincent B. Chen, Jeffrey J. Headd, Nigel W. Moriarty, Jane S. Richardson, David C. Richardson, Alexandre Urzhumtsev, Peter H. Zwart, Paul D. Adams

**Affiliations:** a Lawrence Berkeley National Laboratory, One Cyclotron Road, MS64R0121, Berkeley, CA 94720, USA; b Biochemistry Department, Duke University Medical Center, Durham, NC 27710, USA; c IGBMC, CNRS-INSERM-UdS, 1 rue Laurent Fries, BP 10142, 67404 Illkirch, France; d Université Nancy: Département de Physique – Nancy 1, BP 239, Faculté des Sciences et des Technologies, 54506 Vandoeuvre-lès-Nancy, France; e Department of Bioengineering, University of California Berkeley, CA 94720, USA

**Keywords:** *PHENIX*, Protein Data Bank, data quality, model quality, structure validation, *R* factors

## Abstract

*phenix.model_vs_data* is a high-level command-line tool for the computation of crystallographic model and data statistics, and the evaluation of the fit of the model to data. Analysis of all Protein Data Bank structures that have experimental data available shows that in most cases the reported statistics, in particular *R* factors, can be reproduced within a few percentage points. However, there are a number of outliers where the recomputed *R* values are significantly different from those originally reported. The reasons for these discrepancies are discussed.

## Abstract 2

Application of *phenix.model_vs_data* to the contents of the Protein Data Bank shows that the vast majority of deposited structures can be automatically analyzed to reproduce the reported quality statistics. However, the small fraction of structures that elude automated re-analysis highlight areas where new software developments can help retain valuable information for future analysis.

## 1. Introduction

A tool for quickly obtaining an overview of crystallographic model quality, diffraction data statistics and indicators of the fit of the model to the data is very helpful at all stages of structure solution and validation. Such a tool requires the application of multiple complex and diverse algorithms. For example, it must be capable of processing different representations of atomic displacement parameters including translation–libration–screw (TLS) information (Schomaker & Trueblood, 1968 ▶), analysis of both X-ray and neutron data and data collected from twinned crystals, as well as handling novel ligands or nonstandard residues, Protein Data Bank (PDB; Bernstein *et al.*, 1977 ▶; Berman *et al.*, 2000 ▶) files with multiple models or alternative conformations, and the many reflection data file formats currently in use. We have developed a new program, *phenix.model_vs_data*, which is a part of the *PHENIX* project (Adams *et al.*, 2002 ▶, 2010 ▶). This program automatically handles a large variety of inputs with minimal user intervention. The high degree of automation and ease of use make it possible to routinely run *phenix.model_vs_data* for quick but comprehensive evaluations with results presented in a concise form.

We have tested *phenix.model_vs_data* extensively by automatically processing all PDB models (Joosten, Womack *et al.*, 2009 ▶; Joosten, Salzemann *et al.*, 2009 ▶) for which experimental data are available. Here we describe this new tool and illustrate its use. Running *phenix.model_vs_data* across the whole PDB database we observe that there are a number of entries for which the reported statistics are not reproduced; the reasons for this are discussed, highlighting the difficulties that can be encountered in reproducing statistical quality metrics.

## 2. Methods

### 2.1. *phenix.model_vs_data* input and output

*phenix.model_vs_data* reads a model file in PDB format (Bernstein *et al.*, 1977 ▶; Berman *et al.*, 2000 ▶) and a file with experimental, reduced reflection data. For example,
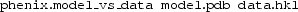
Many commonly used reflection file formats are supported directly, such as *MTZ* (*CCP4* suite; Collaborative Computational Project, Number 4, 1994 ▶), *X-plor*/*CNS* (Brünger *et al.*, 1998 ▶), *SHELX* (Sheldrick, 2008 ▶) and *SCALEPACK* (Otwinowski & Minor, 1997 ▶). If multiple reflection data sets are detected, the user is prompted to specify which data array to use. It is also possible to pass multiple reflection files, for example a file with experimental data and a separate file with free-*R* flags (Brünger, 1992 ▶).

**Figure 1:**
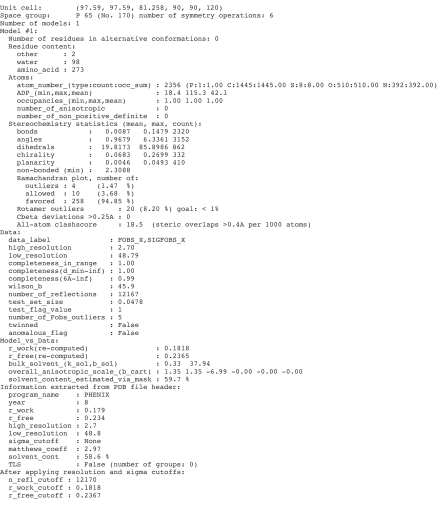
Example *phenix.model_vs_data* output (for PDB entry 3dcv). Model information includes composition and geometry statistics. Data information includes completeness in resolution shells. Model-to-data fit information includes *R* factors calculated for the whole set of structure factors using an optimized bulk-solvent model, anisotropic scaling, and TLS and twinning if applicable. *R* factors are also recalculated after applying the resolution limits and σ cutoffs reported in the PDB header.

The *phenix.model_vs_data* output contains four main sections: (1) model validation statistics, (2) data statistics, (3) a fit of the model to the diffraction data and (4) additional information extracted from the PDB file header if available. The output is plain text (Fig. 1 ▶). The statistics can be inspected from the output to the screen, or from the Python script level by accessing the corresponding attributes of the returned *phenix.model_vs_data* object.

If requested, an electron (for X-ray data) or nuclear (for neutron data) density map can be created by specifying a map type. Supported are regular or maximum-likelihood weighted maps (σ_A_ map; Read, 1986 ▶; Urzhumtsev *et al.*, 1996 ▶) such as 2*mF*_obs_–*DF*_calc_, 3*F*_obs_–2*F*_calc_, anomalous difference maps, average kick maps (Pražnikar *et al.*, 2009 ▶) and the replacement of missing *F*_obs_ with *DF*_calc_ [for more details see Murshudov *et al.* (1997 ▶) and Adams *et al.* (2010 ▶), and references therein]. The output file is in *MTZ* format and contains Fourier map coefficients that can be readily displayed in the *COOT* program (Emsley & Cowtan, 2004 ▶).

Another option is the computation of map correlation coefficients. The two maps that are correlated are the 2*mF*_obs_–*DF*_calc_ map and the *F*_calc_ map. The latter is computed as the Fourier transform of only the *F*_calc_ for which there are corresponding experimental observations available to account for the effects of finite resolution and possible incompleteness of the experimental data. Depending on the resolution of the input data, the correlation coefficients are shown per atom or per residue. Since the correlation alone is not always conclusive, density values of normalized (‘sigma-scaled’) 2*mF*_obs_–DF_calc_ and *mF*_obs_–*DF*_calc_ maps are shown along with each correlation coefficient (the maps are normalized using the standard deviation, as is common practice). This facilitates quick assessment of local model-to-density fits characterized by regions with a poor map correlation and low 2*mF*_obs_–*DF*_calc_ density values or high absolute densities in the *mF*_obs_–*DF*_calc_ map.

### 2.2. *phenix.model_vs_data* algorithms

*phenix.model_vs_data* makes extensive use of the *CCTBX* library (Grosse-Kunstleve *et al.*, 2002 ▶). For example, input PDB files are processed with the comprehensive PDB library implemented in the *CCTBX*. The Monomer Library (Vagin & Mur­shudov, 2004 ▶; Vagin *et al.*, 2004 ▶) is used to obtain geometry restraints (bond, angle, dihedral, chirality, planarity and nonbonded restraints). If an input model contains residues not defined in the Monomer Library, for example a novel ligand or nonstandard residue, *phenix.ready_set* (N. W. Moriarty, unpublished), which uses *eLBOW* (Moriarty *et al.*, 2009 ▶) internally, is used to automatically generate suitable restraints.

The second part of the model-quality section contains summary statistics similar to those generated by the MolProbity web site (Davis *et al.*, 2007 ▶; Chen *et al.*, 2010 ▶), by using the tools integrated into *PHENIX. phenix.ramalyze* is used to compute the number of Ramachandran outliers, as well as favored and allowed residues (Lovell *et al.*, 2003 ▶), and *phenix.cbetadev* is used to compute the number of residues with >0.25 Å deviation from ideal Cβ positions (Lovell *et al.*, 2003 ▶). *phenix.rotalyze* calculates the percent sidechain rotamer outliers (Lovell *et al.*, 2000 ▶). *phenix.reduce* and *phenix.probe* are used to add H atoms and calculate the all-atom clashscore (Word *et al.*, 1999 ▶).

*phenix.xtriage* (Zwart *et al.*, 2005 ▶) is used to detect possible twinning (see, for example, Parsons, 2003 ▶; Helliwell, 2008 ▶). In the presence of possible twin laws, the *R* factors are computed without any twin law and then by taking each twin law into account. The twin-related calculations can be relatively time consuming, but provide a more robust basis for deciding if twinning needs to be included.

If a model was previously refined using TLS parameters, the ATOM and ANISOU records in the coordinate section of the PDB file may contain either total or residual atomic displacement parameters, depending on the refinement program used. The nature of the atomic displacement parameters is often not clear from the TLS information stored as REMARK records in the PDB file header. Therefore two alternatives are tested: *R* factors are computed assuming (i) total atomic displacement parameter values and (ii) residual atomic displacement parameter values in the coordinate section of the PDB file. The outcome with the lowest *R* factor is taken to be correct. Typical *R*-factor differences are 2–10%. The *phenix.tls* (P. V. Afonine, unpublished) module in the *CCTBX* is used to extract the TLS information (selections, origins, matrices) from the PDB file header. Two commonly used formats are automatically distinguished: *phenix.refine* (Afonine *et al.*, 2005*a* ▶) and *REFMAC* (Murshudov *et al.*, 1997 ▶).

*R* factors are computed after performing bulk-solvent correction and anisotropic scaling as described by Afonine *et al.* (2005*b* ▶). The Wilson *B* factor shown in the output is computed using a likelihood procedure (Zwart *et al.*, 2005 ▶). Reflection data outliers are automatically detected (Read, 1999 ▶) and removed from subsequent calculations. The number of outliers is reported in the output.

*phenix.model_vs_data* also supports PDB files with multiple models [see, for example, Burling & Brünger (1994 ▶), Levin *et al.* (2007 ▶), Terwilliger *et al.* (2007 ▶), and references therein]. In addition a list of PDB files can be given as input, facilitating the computation of statistics for very large structures that are currently typically split across multiple files in the PDB.

## 3. Running *phenix.model_vs_data* for entries in the PDB archive

The *phenix.model_vs_data* program has been thoroughly tested by analyzing all PDB entries for which experimental structure factors are available. This was performed in two steps: first the *phenix.cif_as_mtz* tool (P. V. Afonine, unpublished) was used to extract and convert all mmCIF structure factor data files into *MTZ* format (structure factors, σ values and free-*R* flags). Then *phenix.model_vs_data* was run using the generated *MTZ* files with the associated coordinate files. The conversion of CIF format reflection data automatically distinguishes between structure factor intensities or amplitudes, as well as X-ray or neutron data. If possible, the algorithm automatically extracts the free-*R* flags.

**Figure 2:**
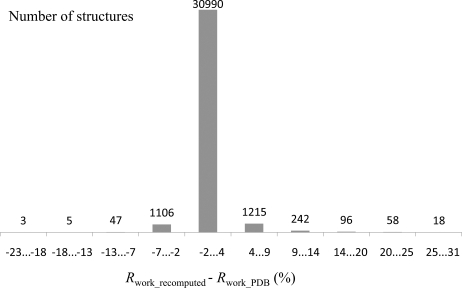
Histogram of differences between *R*_work_ reported in the PDB file header and the value calculated with *phenix.model_vs_data*. Resolution and σ cutoffs were applied in the calculation if available.

The result of analyzing the whole PDB yielded a wealth of useful information currently not always present in PDB depositions: twinning diagnostics, bulk-solvent and scale parameters (Afonine *et al.*, 2005*b* ▶), number of reflection outliers, MolProbity statistics, and Wilson *B* factors. For a number of structures we observed significant discrepancies between the archived metrics (*e.g. R* factors) and their recomputed values. Fig. 2 ▶ shows a histogram of the differences between reported *R*_work_ (as found in the PDB file header) and the recomputed value. In the following section we discuss the factors that can lead to differences in the *R* factors. A somewhat similar discussion is presented by Kleywegt *et al.* (2004 ▶). We note that numerical considerations, such as the method used to calculate structure factors (*i.e.* direct *versus* fast Fourier transformation) have little impact on the results and the difference between *R* factors computed using the different methods is typically less than 0.01%.

### 3.1. Reasons for *R*-factor discrepancies

#### 3.1.1. Missed twinning

Our analysis of the PDB indicates that approximately 3% of all crystal structures are affected by twinning [see Lebedev *et al.* (2006 ▶) for the results of a similar survey of the PDB]. In at least 120 cases, taking twinning into account reduced the *R* factors by 5–20% points.

#### 3.1.2. Variations in bulk-solvent and anisotropic scaling model and related parameters

There are two bulk-solvent models generally used in crystallographic software. One is based on the Babinet principle and is used in the *SHELXL* (Sheldrick, 2008 ▶) and *TNT* (Tronrud, 1987 ▶) programs. The second is a mask-based method based on the flat bulk-solvent model (see Jiang & Brünger, 1994 ▶, and references therein) and is used in programs such as *CNS*, *REFMAC* and *phenix.refine*. In addition, this correction is typically convoluted with overall anisotropic scaling of the diffraction data. There are two different approaches used to perform this anisotropic scaling: using an exponential function (Sheriff & Hendrickson, 1987 ▶; Murshudov *et al.*, 1998 ▶; used in *CNS*, *REFMAC* and *phenix.refine*) or using a polynomial (Parkin *et al.*, 1995 ▶; Usón *et al.*, 1999 ▶; used in *SHELXL*). The mask-based bulk-solvent model has been shown to be superior (Jiang & Brünger, 1994 ▶) and recent methods have been developed to increase the stability of its calculation in combination with anisotropic scaling (Fokine & Urzhumtsev, 2002 ▶; Afonine *et al.*, 2005*b* ▶; Brünger, 2007 ▶). Clearly, recalculation of *R* factors using different bulk-solvent and anisotropic scaling algorithms from those originally used will most likely result in differences. Table 1 ▶ illustrates, for a few selected structures taken at different resolutions, how large the deviations can be.

#### 3.1.3. Missing anisotropic atomic displacement parameters

We observed 14 structures at a resolution higher than 1.0 Å that had all isotropic atomic displacement parameters. For these structures the recomputed *R* factors are several percentage points higher than those reported (see Table 2 ▶ for an example). A review of the literature indicated that at least five of these structures were refined using anisotropic atomic displacement parameters.

#### 3.1.4. Nonphysical anisotropic atomic displacement parameters

To make physical sense, a symmetric matrix representing anisotropic atomic displacement parameters has to be positive definite. We observed several hundred entries with negative-definite anisotropic displacement parameters. The impact on *R* factors depends on the percentage of such atoms in a structure. Considering all cases we observe an average *R*-factor increase of ∼2.5% points, and in the worst case changes of 10% and more. Zero atomic displacement parameter values for H atoms (see §[Sec sec3.1.5]3.1.5) also fall into this category.

#### 3.1.5. Missing H atoms

**Figure 3:**
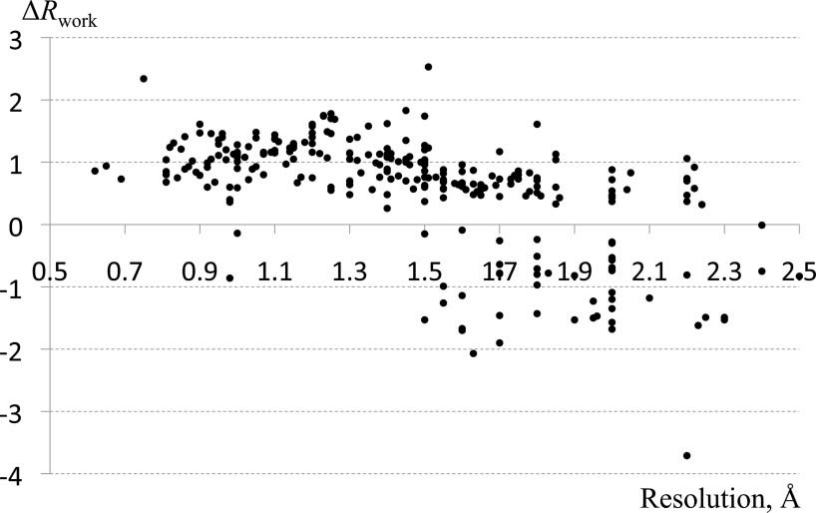
Differences between *R*_work_ computed for the original structures with H atoms and the same structures after removal of the H atoms, shown as function of resolution. See §[Sec sec3.1.5]3.1.5 for details.

**Figure 4:**
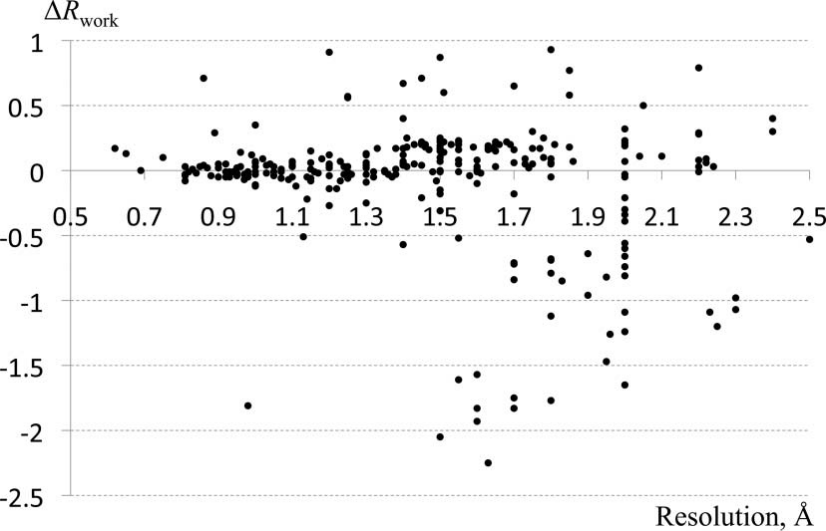
Differences between *R*_work_ values (shown as function of resolution) computed for structures without H atoms and the same structures with restored H atoms based on ideal geometry. The atomic displacement parameter and occupancy of each restored H atom was set to be identical to those of the bonded atom. See §[Sec sec3.1.5]3.1.5 for details.

Analysis of deposited structures indicates that even if H atoms were used in refinement (*e.g.* using a riding model) they are often removed prior to structure deposition. To assess the impact of removing H atoms we selected 275 deposited structures that contain H atoms. Fig. 3 ▶ shows the difference between *R*_work_ factors computed using the original structures and those with all H atoms removed. The contribution from the H atoms is significant, ranging from approximately 0.5 to 2.0 points in *R*_work_, and is essentially independent of resolution. Those structures where removal of the H atoms leads to a decrease in *R*_work_ (*i.e.* negative differences) typically have nonphysical parameters (*e.g.* atomic displacement parameter values of zero for all H atoms). We then assessed the impact of adding H atoms back to those 275 structures. We restored the H atoms using ideal parameters and recomputed the *R* factors. Our observation is that the recomputed *R* factors do not match the original ones, as shown in Fig. 4 ▶. There are a number of reasons for this: different programs may use different libraries to determine the H-atom positions, for example placing H atoms at a nuclear position derived from neutron scattering experiments (Allen, 1986 ▶) or placing them at a shorter distance where the electron density peak is truly observed (as it is implemented in *SHELXL*). The assignment of the H-atom displacement parameters further complicates the calculation. H atoms can inherit the exact atomic displacement parameters of the atoms to which they are bound, or they can take this value multiplied by a factor between 1.0 and 1.5 (see the *SHELXL* manual, for example). At subatomic resolution the H-atom atomic displacement parameters may have been refined to unique values for each atom.

#### 3.1.6. Missing water molecules

We observed a number of structures refined at resolutions better than 2 Å that do not possess any solvent atoms and for which the recalculated *R* factors are different from those originally reported. We selected a few such structures and automatically processed them with *phenix.refine* in order to add water atoms and then recompared *R* factors. Table 3 ▶ summarizes the results. The table suggests that the difference between published and recomputed *R* factors is due to missing solvent atoms. In many cases the differences in solvent structure are small (a few missing water molecules), while in other cases the absence of water molecules results in a very large discrepancy (*e.g.* structure 1ejg).

#### 3.1.7. The use of very high resolution refinement methods: multipolar refinement and interatomic scatterers

At subatomic resolution (better than ∼1 Å) a multipolar (Hansen & Coppens, 1978 ▶) or an interatomic scattering model (Afonine, 2004 ▶, 2007 ▶) can be used to model residual bonding density that is typically visible at such resolutions. Currently, there is no mechanism in the PDB file format to preserve this information, and therefore the *R*-factor statistics obtained in such a refinement cannot be reproduced from the deposited structure. An example is 1ejg, a structure refined at 0.54 Å resolution using multipolar methods.

#### 3.1.8. Structures refined using the TLS model

When TLS refinement is used, the total atomic displacement parameter is typically approximated by the sum of three contributions: the residual atomic displacement parameter representing local atomic vibrations, the component representing the rigid-body displacements modeled through TLS, and the component representing lattice vibrations, which is usually modeled as part of the overall anisotropic scaling.

There are at least two types of PDB files where the TLS information is represented differently: entries where each atom participating in a TLS group has its total atomic displacement parameter reported (for example, structures refined with *phenix.refine*) and entries where only residual atomic displacement parameters are reported for each atom and the TLS component is stored as TLS matrices in the file header (typically, structures refined with *REFMAC*). To recompute the *R* factors, it is essential that the displacement information for each atom be correctly retrieved from the PDB file and the total atomic displacement parameter for each used. This in turn makes it vital for the structures where residual atomic displacement parameters are reported that the TLS information, namely TLS origin, values of the TLS matrices and the TLS group definition, can be correctly extracted from the PDB file header.

As of December 2009, there are 8278 structures (out of a total of 62 305) that contain TLS information. For 730 of these entries the TLS information cannot be correctly extracted. The typical problems in TLS records can be classified into three categories: (*a*) missing, empty, duplicate, ambiguous or syntactically incorrect TLS group selections; (*b*) missing or incorrectly defined TLS group origins; (*c*) problems with the TLS matrices (for example, incorrect formatting).

#### 3.1.9. Other factors

Other possible reasons for discrepancies between reported and recalculated *R* factors are as follows:

(*a*) Absence of test set (cross-validation) flags, so *phenix.model_vs_data* uses all (work and test) reflections to compute the *R* factor.

(*b*) Some programs allow refinement of *f*′ and *f*′′ for anomalous scatterers. However, the refined *f*′ and *f*′′ values are typically not preserved in the PDB file header, and therefore they are not used in structure factor calculations.

(*c*) Various manipulations on *F*_obs_, such as removing outliers and applying anisotropic corrections.

(*d*) Incomplete, missing or incorrect information in the file header about data cutoffs used in statistics calculation (by resolution, σ).

(*e*) Running a final refinement against all data (instead of excluding the *R*-free set) before deposition.

### 3.2. Special cases

Most crystallographic entries in the PDB are derived from X-ray diffraction data, and are represented as a single atomic model. However, there are special cases, which constitute only a very small fraction of all the entries: structures determined using neutron data, multiple model entries or extremely large structures. While most crystallographic software can seamlessly handle single-model X-ray structures (given that the appropriate libraries for nonstandard items, such as ligands, are provided), handling these special cases can be a challenging problem. The *phenix.model_vs_data* program was developed to handle such special cases with the results described below.

#### 3.2.1. Multiple model entries

There are 125 crystal structures in the PDB that are represented by multiple models; 114 of them have experimental data available. Among those 114 data sets, nine contain Miller indices that are not unique under the symmetry with several hundreds of redundant reflections, and 49 files contain multiple data sets, making automated interpretation uncertain. Table 4 ▶ shows the summary of running *phenix.model_vs_data* for the remaining 56 entries. For 28 of these the recalculated *R* factor was within 5% of the reported value. Seven entries (2g0v, 2g0x, 2g0z, 2g10, 2g11, 2g12, 2g14) report *R* values obtained after *difference refinement* (Terwilliger & Berendzen, 1995 ▶) that reflect the agreement between model differences and data differences. Therefore it is not possible to reproduce these deposited *R* values; however, the computed *R* factors are all within the 17.3–18.5% range. Ten structures (1yrq, 1zev, 2ce2, 2cl6, 2cl7, 2clc, 2cld, 2evw, 2gn0, 3cmy) have recalculated *R* factors about five percentage points higher than the reported values. This is because these structures were subject to TLS refinement, but the TLS selections in the PDB file headers do not unambiguously define the TLS groups, making it impossible to reproduce the total atomic displacement parameters of the affected atoms (for these structures only residual atomic displacement parameters are present in ATOM records). The 2ull entry has high recomputed *R* factors. The corresponding PDB file contains 16 models, and each protein atom within each model has the occupancy of 0.06, making the total occupancy ∼0.96 (16 × 0.06). This should not pose problems if the overall occupancies are identical for each model and the number of models is less than 100: the overall scale factor will account for this numerical rounding. However, for PDB entry 2ull the solvent structure is identical for each of 16 models, but unlike the occupancies of the protein atoms those of the solvent atoms are not scaled to sum to one. We consider this the main reason for the *R*-factor mismatch.

#### 3.2.2. Large structures spread across multiple files

There are 52 structures in PDB that are split across multiple files; 45 of them are crystal structures. Of these, 40 crystallographic structures have the experimental data deposited. Three of the 40 entries were excluded from tests because we could not extract the data (2zuo, 2zv4, 2zv5), or the data files are not unique under symmetry (1jyy, 1jyz, 1jz0, 1jz1). *phenix.model_vs_data* could reproduce the *R* factors for the remaining 37 (results not shown).

#### 3.2.3. Structures determined using neutron data

Currently 32 structures in the PDB were determined using neutron diffraction data, 26 of which have experimental structure factor data available. Table 5 ▶ summarizes the *R*_work_ and *R*_free_ values extracted from the PDB file headers and those recomputed using *phenix.model_vs_data*. In only six cases out of the total of 26 did the recomputed *R*_work_ not match the published values. In four of these cases this is because *R*_work_ was not available in PDB file header. However, we still observed situations that make it challenging to recompute the *R* values:

(*a*) The sum of occupancies for exchangeable H/D sites (see, for example, Niimura *et al.*, 2006 ▶) is smaller than 1.

(*b*) Incorrect or missing information in the PDB file header, such as missing *R* factors or σ cutoff values.

(*c*) H/D exchange is not modeled or is incompletely modeled. For example, the molecule is fully deuterated but the corresponding PDB file contains all H atoms instead of D atoms. In a number of cases only a small fraction of the potentially exchangeable sites are modeled.

(*d*) In some cases the reflection data intensities are mis-labeled as amplitudes or *vice versa*. We note that this problem is not limited to neutron diffraction data.

(*e*) Atoms with negative occupancies.

(*f*) Atoms with an undefined scattering type, *e.g.* labeled as *X*.

## 4. Conclusion

The output of the *phenix.model_vs_data* program is designed to enable easy validation of model and data files, and of commonly reported model/data statistics, in particular as found in PDB file headers. To assure a high degree of automation and robustness, the *phenix.model_vs_data* program is routinely tested by processing all PDB entries for which experimental data are available. The statistics generated are actively used in the development of the *PHENIX* system. An example of an application of this database is the *POLYGON* program (Urzhumtseva *et al.*, 2009 ▶), which provides a concise graphical comparison of model quality measures with similar entries found in the PDB.

Application of *phenix.model_vs_data* to the contents of the PDB shows that the vast majority of deposited structures can be automatically analyzed to reproduce the reported quality statistics. However, there remain a small fraction of structures that elude automated re-analysis. These highlight areas where new developments in structure deposition tools and refinement software can help retain valuable information for future analysis.

*phenix.model_vs_data* is available as part of the *PHENIX* package, which can be obtained from http://www.phenix-online.org.

## Figures and Tables

**Table 1:** Comparison of published (column 3) *R* factors and solvent parameters with those recomputed using default parameters (column 4), recomputed using published values of *k*_sol_ and *B*_sol_ (column 5), and recomputed using slightly different values of *r*_shrink_ and *r*_solv_ (those used in *REFMAC*; last column) All values were recomputed with *PHENIX*.

		Published (from PDB file header)		Computed with *r*_shrink_ = 0.9, *r*_solv_ = 1.11		Recomputed with published *k*_sol_/*B*_sol_	Recomputed with *r*_shrink_ = 0.8, *r*_solv_ = 1.2	
PDB code	Resolution (Å)	*R*_work_/*R*_free_	*k*_sol_/*B*_sol_	*R*_work_/*R*_free_	*k*_sol_/*B*_sol_	*R*_work_/*R*_free_	*R*_work_/*R*_free_	*k*_sol_/*B*_sol_
1jvx	2.5	23.2/30.4	0.55/132.1	23.0/29.8	0.32/60.0	23.3/30.5	23.8/30.4	0.31/60.0
1jzb	2.8	23.3/27.7	0.58/122.4	22.7/24.6	0.28/25.9	23.1/27.1	22.6/24.6	0.29/21.5
1kk7	3.2	25.9/31.3	0.31/162.0	24.7/28.1	0.20/60.0	25.4/29.2	24.6/28.1	0.20/60.0
1r30	3.4	25.6/30.0	0.34/136.6	22.7/26.1	0.31/80.0	23.2/26.9	22.6/26.3	0.31/80.0
1tve	3.0	28.9/36.3	0.32/108.7	27.0/35.0	0.33/46.1	27.4/35.5	26.9/35.2	0.32/43.4
3cf1	4.4	22.9/28.6	0.30/179.2	25.3/29.0	0.32/198.4	25.5/29.3	26.2/29.9	0.31/197.7

**Table 2:** Example of structures where the original anisotropic atomic displacement parameters are missing and the corresponding PDB files contain only isotropic atomic displacement parameters Columns 3 and 4 show the published and recomputed *R* factors. See §[Sec sec3.1.3]3.1.3 for details.

PDB code	Resolution (Å)	*R*_work_	*R*_work_ recomputed
352d	0.95	15.2	20.8
1brf	0.95	13.2	17.1
1dj6	1.00	16.5	19.2
2fn3	1.00	12.8	17.0
1pjx	0.85	12.1	16.6
1q6z	1.00	12.2	17.2
1ucs	0.62	13.7	17.6

**Table 3:** Example of PDB entries with missing water molecules See §[Sec sec3.1.6]3.1.6 for details.

	*R*_work_/*R*_free_			
PDB code	Published	Recomputed with *phenix.model_vs_data*	Water added with *phenix.refine*	Number of added water molecules
1kel	19.9/25.8	26.4/27.2	17.4/21.7	648
1nko	27.7/30.1	27.1/29.3	19.8/22.1	108
1p4k	18.2/22.0	22.3/25.1	15.3/19.8	603
1r3f	22.8/25.7	25.0/26.0	18.8/23.0	240
1rh9	18.2/20.5	25.5/25.9	18.7/21.3	508
1wou	21.9/22.9	23.6/24.0	19.0/22.9	42
1xxs	16.6/24.7	22.1/24.5	18.8/22.6	117
2jjf	16.6/18.5	21.3/22.1	15.3/17.6	260
2ou9	15.9/22.0	28.4/29.8	19.1/21.4	312
2z1y	18.0/21.7	24.1/24.1	16.5/19.6	1051
3d9z	14.5†/19.0	19.9/20.5	15.0/17.8	199
3fy3	14.9/20.3	24.0/26.3	18.4/23.0	185
6msi	21.5†/28.0†	23.3/24.1	17.9/22.1	48
1ejg	9.0/9.4	20.8/20.7	8.3/8.6	128

† The corresponding *R* factors were not available in PDB file header and the values were extracted from the corresponding publications.

**Table 4:** Crystal structures represented by multiple models *R*_work_ and *R*_free_ as extracted from the PDB file headers (second column) and as recalculated using *phenix.model_vs_data* (third column). (n.a.: not available.)

	*R*_work_ and *R*_free_			*R*_work_ and *R*_free_	
PDB code	PDB file header	Recomputed with *model_vs_data*	PDB code	PDB file header	Recomputed with *model_vs_data*
1gu8	23.0/25.6	23.0/25.7	2g0v	5.1/5.4	18.5/n.a.
1htq	20.4/22.3	20.7/n.a.	2g0x	5.5/5.3	18.5/n.a.
1l2g	27.8/29.7	25.7/28.7	2g0z	5.8/7.0	18.4/n.a.
1mz0	15.0/17.3	14.6/16.7	2g10	4.5/4.9	17.3/n.a.
1n6j	24.3/26.8	28.5/31.2	2g11	5.1/5.7	17.4/n.a.
1ohh	23.2/28.0	21.7/n.a.	2g12	5.3/6.2	17.4/n.a.
1ot6	14.4/16.1	14.6/n.a.	2g14	5.1/5.8	17.3/n.a.
1ot9	13.4/16.1	13.5/n.a.	2g32	23.9/25.8	25.1/27.3
1t3n	26.5/28.6	25.6/28.0	2gn0	18.8/22.2	23.1/25.9
1u0c	21.4/27.7	28.6/n.a.	2gpm	n.a./27.0	24.8/33.0
1u0d	21.7/25.7	37.8/38.5	2gq4	n.a./27.0	25.1/28.4
1vjm	25.2/29.8	24.7/29.3	2gq5	n.a./31.8	26.5/31.7
1wte	17.1/22.3	21.2/26.3	2gq6	n.a./29.5	27.4/28.5
1x0i	23.8/28.2	25.2/28.9	2gq7	n.a./31.0	24.8/31.2
1yk0	24.0/28.4	23.5/23.8	2grz	10.6/10.9	56.9/58.8
1yrq	17.1/22.0	22.4/26.0	2j9j	14.2/19.1	15.3/n.a.
1zbl	21.7/25.3	26.0/28.2	2je4	14.3/18.4	21.4/n.a.
1zev	21.8/27.9	29.0/33.1	2ntw	15.3/19.5	14.4/n.a.
1zy8	20.8/27.6	20.9/27.1	2q3m	15.7/21.7	15.7/21.2
2aaz	29.0/30.5	27.8/29.4	2q3o	18.0/23.5	17.9/23.1
2ce2	14.4/16.3	21.8/23.3	2q3p	18.2/22.4	18.1/21.9
2cl6	14.6/18.6	23.8/27.4	2q3u	13.5/17.1	14.3/17.4
2cl7	14.8/17.0	20.3/23.4	2ull	16.5/19.2	50.1/n.a.
2clc	14.9/18.0	23.7/27.0	2vtu	27.2/31.0	30.7/26.6
2cld	14.9/17.6	21.9/24.8	3c5f	22.4/26.3	22.2/26.1
2d6b	18.2/21.3	17.3/n.a.	3cmy	17.2/21.3	22.4/25.1
2e1c	20.6/23.0	31.1/31.4	3cye	19.3/23.1	18.1/22.0
2evw	15.6/23.6	20.9/23.6	406d	26.2/29.4	33.6/35.8

**Table 5:** Crystal structures solved using neutron data *R*_work_ and *R*_free_ as extracted from PDB file header (second column), and as recalculated using *phenix.model_vs_data* (third column).

	*R*_work_ and *R*_free_	
PDB code	PDB file header	Recalculated with *phenix.model_vs_data*
1c57	27.0/30.1	30.0/33.7
1cq2	16.0/25.0	32.7/32.8
1iu6	20.1/22.8	20.6/23.2
1l2k	20.1/23.8	19.9/23.3
1v9g	22.2/29.4	24.6/30.4
1vcx	18.6/21.7	18.5/21.4
1wq2	22.9/28.9	27.8/31.3
1wqz	25.2/27.4	24.0/30.3
1xqn	26.6/32.0	35.3/35.7
2dxm	19.7/26.0	20.4/26.7
2efa	21.6/29.1	24.5/28.9
2gve	27.1/31.9	25.0/30.1
2inq	n.a./23.3	20.8/24.8
2mb5	n.a.	23.7/n.a.
2r24	25.7/29.1	25.6/29.1
2vs2	21.9/28.1	23.1/22.7
2yz4	27.9/31.2	28.1/31.4
2zoi	19.2/21.9	19.8/22.1
2zpp	22.1/26.0	23.1/27.4
2zye	19.3/22.2	19.4/22.0
3byc	26.4/31.5	27.1/28.6
3cwh	23.7/28.8	23.9/23.1
3hgn	19.6/21.6	19.6/21.5
3ins	18.2/n.a.	19.3/n.a.
5pti	n.a.	18.7/n.a.
5rsa	n.a.	18.3/n.a.
